# DPOAE growth function in schoolchildren with impaired temporal ordering skills

**DOI:** 10.1590/2317-1782/e20240071en

**Published:** 2025-02-10

**Authors:** Patricia Kimiko Kumagai, Seisse Gabriela Gandolfi Sanches, Renata Mota Mamede Carvallo

**Affiliations:** 1 Faculdade de Medicina, Universidade de São Paulo – USP - São Paulo (SP), Brasil.; 2 Departamento de Fisioterapia, Fonoaudiologia e Terapia Ocupacional, Faculdade de Medicina, Universidade de São Paulo – USP - São Paulo (SP), Brasil.

**Keywords:** Otoacoustic Emissions Spontaneous, Auditory Perception, Hearing Tests, Hearing, Child

## Abstract

**Purpose:**

To investigate whether the cochlear responses of a group of children with normal temporal ordering tests would be different from those children with abnormal results in the same tests.

**Methods:**

25 children aged 8 to 13 years participated in the study, all with normal range pure-tone audiometry thresholds, type A tympanometry and distortion product otoacoustic emissions (DPOAE) present in both ears. Of these, 13 children formed the Study Group and 12 the Control Group. The Study Group differed from the Control Group by presenting changes in temporal auditory tests. In addition to the tests to verify the inclusion criteria, the DPOAE growth function were recorded for three different f2 frequencies, respectively 2002, 3003 and 4004 Hz. The stimuli were presented at level f2 (L2) from 20 to 65 dB SPL in steps of 5 dB and the stimulus level f1 (L1) followed the formula: L1=0.4L2+39 dB. The data were analyzed statistically, adopting a significance level of 5%.

**Results:**

The groups did not differ in relation to conventional DPOAE values (DP-Gram). The Study Group differed from the Control Group by exhibiting both the threshold (p=0.034) and the higher slope (p=0.043) in the 2000 Hz DPOAE growth.

**Conclusion:**

Children with alterations in temporal ordering tests require greater intensity to reach the DPOAE threshold at a frequency of 2000 Hz when compared to children without complaints, also presenting a more linear cochlear amplification at this same frequency, indicated by the increase in the value of slope.

## INTRODUCTION

One of the procedures to evaluate the function of the Outer Hair Cells (OHC) present in the Organ of Corti is the assessment of evoked otoacoustic emissions^(1)^. Otoacoustic emissions can be evoked by different stimuli, which also generates different responses that can aid the audiological diagnosis as well as the identification of subtle alterations in the cochlear function^(2-4)^.

The Distortion Product Otoacoustic Emissions (DPOAE) are triggered by the exposure to two pure tones with different frequencies, namely F1 and F2, where F2’s frequency is higher than F1’s. The cochlear response is therefore the result of the interference between these two pure tone stimuli, and the distortion product that corresponds to 2F1-F2 is most commonly used in the clinical practice^(2)^.

The DPOAE presence indicates a preserved cochlear function, whereas its absence demands a thorough investigation of both the middle and inner ear^(5,6)^. Such emissions are fundamental for the patient’s evaluation, assisting in the identification of mild to profound hearing losses in children, hence attenuating the impairment’s negative impacts on language development and literacy^(2)^.

DPOAE recording is obtained by presenting pure tone pairs at a fixed intensity level for F1 and F2, usually 65dBSPL/55 dBSPL, respectively. In the assessment, the set of reactions evoked by the stimuli generates a graph (DP-gram) comprising the cochlear responses to the investigated pure tone pairs, typically ranging between 1000 and 6000Hz at fixed intensity.

With that being said, in addition to the thresholds obtained using fixed intensities at different frequencies, it is also clinically relevant to know the lowest stimulus intensity (F1 and F2) able to evoke a robust otoacoustic emission^(7-9)^. This is attributable to the fact that, although different cochleae may show similar DP-gram responses, they may have different DPOAE thresholds. In other words, distinct cochleae may respond similarly to an average intensity level (65/55 dB) while presenting different DPOAE thresholds. This characteristic may be useful to differentiate cochlear function aspects among people who, regardless of their normal auditory thresholds, present different hearing complaints. In order to investigate the DPOAE threshold, the intensity levels of F1 and F2 can be registered in an increasing or decreasing manner, between 20 and 70 dBSPL, targeting the identification of the stimulus’ lowest intensity capable of generating a robust DPOAE^(10,11)^.

This way, it is possible to record the threshold as well as to determine the growth curve of the DPOAE response in relation to the stimuli increase, creating the graph known as the input/output Growth Function, which provides information on both the DPOAE threshold and the cochlear mechanisms associated with the OHC nonlinearity function^(11,12)^. In normal cochlear function, nonlinear mechanisms provide a higher amplification rate for low-intensity stimuli, thus increasing the sensitivity and frequency selectivity, attributes of a normal auditory function^(13)^. Nonlinear mechanisms promote the compression of cochlear amplification as the stimulus increases^(13,14)^, that is, they decrease the amplification rate for medium intensity sounds. This nonlinear amplification establishes the mechanisms that contribute to the high sensitivity, wide dynamic range and fine-tuning of the mammalian auditory system^(15)^.

Moreover, concepts such as “non-linearity” and “compression” in the cochlear function are closely correlated. A healthy cochlea maintains a linear amplification for low-intensity stimuli, decreases amplification for medium-intensity stimuli (compression), and increases yet again for high-intensity stimuli, where the reduction in amplification for medium intensity stimuli is associated with the cochlear gain compression ratio. The DPOAE allow the evaluation of the compression rate of the cochlear amplifier through its input/output growth functions, and this compression is measured using the slope of the growth curve^(13)^. Further studies have also found a decline in the compression ratio due to aging, as evidenced by the more linear slope associated with older age groups^(7,11,16)^.

The DPOAE Growth Curve threshold is established as the lowest L_2_ intensity able to evokes a robust response. Higher intensity level DPOAE thresholds may be related to the decrease in overall DPOAE response shown in the DP-gram^(5,7,17)^.

The auditory temporal processing impacts the individual's ability to identify changes in the characteristics of sounds, such as frequency, intensity, duration and breaks between stimuli. Auditory perception requires an accurate processing of the sound duration’s structure. One of the tasks involved in auditory processing is responsible for the perception of rapid changes in the stimulus pattern and the integration of information that occurs over time. This perception is called temporal processing. Temporal processing skills encompass: temporal ordering or sequencing, temporal integration or summation, temporal masking and temporal resolution or discrimination^(18)^.

Temporal ordering is responsible for the identification of two or more stimuli presented in sequence during a given period of time, and is also related to the understanding of sound information^(18)^. Some researchers suggest that unsatisfactory reading performances may be associated with an auditory temporal processing disorder, making it harder for children to hear acoustic changes in sounds, which can lead to an impaired speech perception^(19)^ and phonological awareness difficulties. When studying children with reading and writing disorders, Soares et al.^(20)^ observed a correlation between alterations in temporal processing and deficits in phonological awareness.

Considering that slight cochlear changes identified by growth curves affect temporal resolution, one of the subclassifications of temporal processing^(6)^, it is relevant to assess whether it is also possible to identify subtle cochlear alterations regarding temporal ordering difficulties.

Due to the fact that DPOAE growth curves may be an additional tool for the identification of discrete impairments of cochlear function^(7)^, it may be useful in the investigation of peripheral auditory function in children presenting alterations in temporal auditory tests.

Based on the hypothesis that the performance in temporal auditory assessments may be affected by changes in the cochlear function’s compression rates, the objective of this study is to investigate whether the cochlear responses of a group of children with temporal ordering tests within the normal range would be different from those found in children with such altered tests.

## METHODS

The present study was approved by the Institutional Research Ethics Committee, on 09/24/2019, according to Opinion No. 3.631.330 and CAAE No. 20319119.0.0000.0065. All study participants signed the Informed Consent Form as well as the Parental Consent and Child Assent, approving the participation in this research.

The convenience sample of this study consisted of 25 children, between 08 and 13 years old, with 12 assigned as the Control group (5 females and 7 males) and 13 as the Study group (6 females and 7 males). The mean age for the Control group was 9.8 (±1.34) years old and, in the Study group, 10.5 (±1.71) years. The inclusion criteria were: registering tonal thresholds up to 20 dBHL at frequencies from 250 to 8000 Hz; presenting DPOAE responses; and recording a Type A tympanogram with a 226Hz probe. For the Study group children, the presence of a temporal auditory processing impairment was also considered an inclusion criterion, determined by an alteration in the frequency pattern test and/or in the duration pattern test. The participants of the Control group showed no changes in both temporal ordering tests, and the normality standard was adopted for Portuguese speaking Brazilian children^(21)^. Individuals with diagnosed attention disorder or hyperactivity disorder, as well as with complaints and/or diagnosis of neurological or psychiatric disorders were thereby excluded.

All participants underwent external auditory canal examination; tympanometry and acoustic reflex assessment (ipsi and contralateral) with the AT 235h equipment (Interacoustics). Then, pure-tone air-conduction testing was performed using the GSI 61 audiometer (Grason Stadler), with TDH 50P headphones. The frequencies from 0.25 to 8 kHz, in octave intervals, were tested applying the ascending-descending method. Subsequent to the compliance with the inclusion criteria, the participants performed the following temporal processing assessments (children's version, Auditec Saint Louis^(22)^): Frequency Pattern Test (FPT) and Duration Pattern Test (DPT).

After the battery of behavioral tests, the Evoked Otoacoustic Emissions – distortion product (DPOAE) were collected by the ILO 292II V6 OAE Otodynamics equipment, with the patient inside an acoustic booth.

Two methods were favored to obtain the DPOAE: DP-Gram and Growth Curves:

**A-** For the DP-Gram, the stimuli f1 and f2 were presented at the fixed intensity levels of L1 and L2, or 65 dB and 55 dB, respectively, along with f2 frequencies variations ranging from 1001 to 6006 Hz (2 points per octave), with f2/f1 being approximately 1.22. Responses with a Signal-to-Noise ratio greater than 6 dB SPL in relation to the standard deviation of the background noise were considered as present.
**B-** Three DPOAE growth curves were plotted for both ears, selecting a single F2 frequency for each of the curves in the DP Growth function using the ILO V6 program. Both the DPOAE growth curve records and the DPOAE threshold determination were performed for the three different f2 frequencies of 2002, 3003 and 4004 Hz, respectively. Stimuli were presented at an intensity level of f2 (L2) ranging from 20 to 65 dB SPL in 5 dB steps and the stimulus intensity level f1 (L1) varied according to the formula proposed by Kummer et al.^(23)^: L1= 0.4L2+39 dB. The rule used to interrupt the test was determined with the background noise floor established below 10 µPA at all stimulus intensities analyzed herein and DPOAE responses were stabilized (almost no variation between scans). The DPOAE threshold was considered as the lowest intensity in which there was a signal/noise ratio ≥ 03 dB with the two consecutive higher intensities presenting a greater or equal signal/noise ratio. The curve’s grade was evaluated by its slope (software generated measurement) in order to verify the cochlear compression.

The child's comfort was considered throughout all examinations, with the tests being interrupted in case of any signs of discomfort or fatigue exhibited by the participant.

To calculate the 95% confidence intervals, the Bias-Corrected and Accelerated method was used based on 2000 bootstrap samples. The bracketed values in the tables indicate the upper and lower limits of the 95% confidence intervals.

DPOAE responses (signal-to-noise ratio) were evaluated according to the growth curves’ profile, threshold (L2) as well as slope. For the comparison between groups, Student's t-test was used for independent samples (parametric) and Mann-Whitney U-test (non-parametric) was applied when the assumption of normality of data distribution was preserved (p > 0.05, Shapiro-Wilk normality test). Whenever the Student's t-test was used for independent samples in which a violation of the homoscedasticity assumption was observed (p ≤ 0.05, Levene's test), a Welch's correction for heteroscedasticity was applied to calculate the p value. The effect size of the difference between the groups was measured by calculating the coefficient d or r. The statistical significance value adopted was 5% (p ≤ 0.05), using the SPSS Statistics software, version 25.0 (IBM Corp., Armonk, NY, USA).

### Results

The present study analyzed the cochlear responses of children as to the threshold and configuration of the DPOAE growth curve through the slope measurement provided by the ILO 292 II - V6 software (Otodynamics) in students with and without alterations in temporal auditory tests.

The statistical analysis of this study’s data was performed using the sample of 50 ears organized into two groups: Control Group (CG, n = 24) and Study Group (SG, n = 26).

There was no significant difference between the ages of the group (p = 0.320). There was also no significant difference between the groups regarding the hearing thresholds for all frequencies between 250 and 8000 Hz.

The results described in Table 1 demonstrate that there was no statistically significant difference between the groups in relation to the reflection thresholds ranging from 500 to 4000 Hz. Therefore, individuals presenting alterations in the temporal ordering tests recorded acoustic reflex thresholds similar to those without alterations in temporal ordering tests.

**Table 1:** Descriptive values and comparative analysis of the groups in relation to the acoustic reflex thresholds

**Frequency (Hz)**	**Group**	**No**	**Mean**	**SD**	**Median**	**Min.**	**Max.**	**P**	**E.S.**
500	CG	24	93.33	6.70	95.00	75.00	105.00	0.708	0.055
			[90.42, 96.04]		[95.00, 95.00]				
	SG	24	94.17	7.32	95.00	80.00	105.00		
			[91.32, 96.88]		[95.00, 95.00]				
1000	CG	24	92.71	8.84	95.00	75.00	110.00	0.587	0.165
			[89.38, 95.83]		[87.50, 95.00]				
	SG	24	94.17	9.63	95.00	75.00	110.00		
			[90.42, 97.92]		[92.50, 97.50]				
2000	CG	24	91.67	9.96	90.00	75.00	110.00	0.341	0.139
			[87.71, 95.83]		[90.00, 92.50]				
	SG	24	94.58	11.41	95.00	80.00	110.00		
			[90.00, 98.96]		[95.00, 95.00]				
4000	CG	24	97.29	7.07	100.00	80.00	105.00	0.971	0.008
			[94.38, 100.00]		[95.00, 100.00]				
	SG	24	96.04	9.89	100.00	75.00	105.00		
					[95.00, 102.50]				
			[92.08, 100.00]						

Caption: CG = Control Group; SG = Study Group; SD = Standard Deviation; Min. = Minimum; Max. = Maximum; P = p value ; E.S. = Effect Size

Table 2 depicts the measures of central tendency and dispersion (signal-to-noise ratio) of the response from the conventional DPOAE according to the group and frequency. Furthermore, it was found no significant difference between the groups in relation to the conventional DPOAE responses (Table 2).

**Table 2:** Descriptive values and comparative analysis of the groups in relation to the conventional DPOAE response according to the frequency

**DPOAE**	**Group**	**No.**	**Mean**	**SD**	**Median**	**Min.**	**Max.**	**P**	**E.S.**
1001 (S/N – dB)	CG	24	2.38	9.87	2.55	-16.80	20.10	0.291	0.278
			[-1.28, 6.01]		[-0.10, 6.65]				
	SG	26	5.12	8.27	5.65	-10.40	20.60		
			[2.06, 7.97]		[1.05, 7.75]				
1501 (S/N – dB)	CG	24	11.35	11.07	11.00	-9.00	34.00	0.947	0.018
			[7.37, 15.42]		[5.05, 15.85]				
	SG	26	11.55	10.23	12.60	-15.30	28.50		
			[7.60, 15.21]		[6.10, 16.20]				
2002 (S/N – dB)	CG	24	11.68	11.63	13.45	-15.00	32.20	0.518	0.148
			[6.94, 15.97]		[8.20, 16.60]				
	SG	26	13.40	5.89	13.45	1.70	25.40		
			[11.24, 15.44]		[11.55, 15.20]				
3003 (S/N – dB)	CG	24	13.03	10.83	13.45	-15.90	29.00	0.547	0.087
			[8.23, 17.10]		[11.40, 19.10]				
	SG	26	13.40	5.70	12.90	2.60	25.60		
			[11.24, 15.44]		[10.85, 15.30]				
4004 (S/N – dB)	CG	24	17.79	7.22	19.15	5.30	30.00	0.337	0.252
			[15.13, 20.59]		[15.55, 20.10]				
	SG	26	15.97	6.03	16.85	2.60	26.60		
			[13.77, 18.17]		[13.90, 20.10]				

Caption: CG = Control group; SG = Study Group; S/N = Signal-to-Noise ratio; SD = Standard Deviation; Min. = Minimum; Max. = Maximum; P = p value ; E.S. = Effect Size

**Figure 1:**
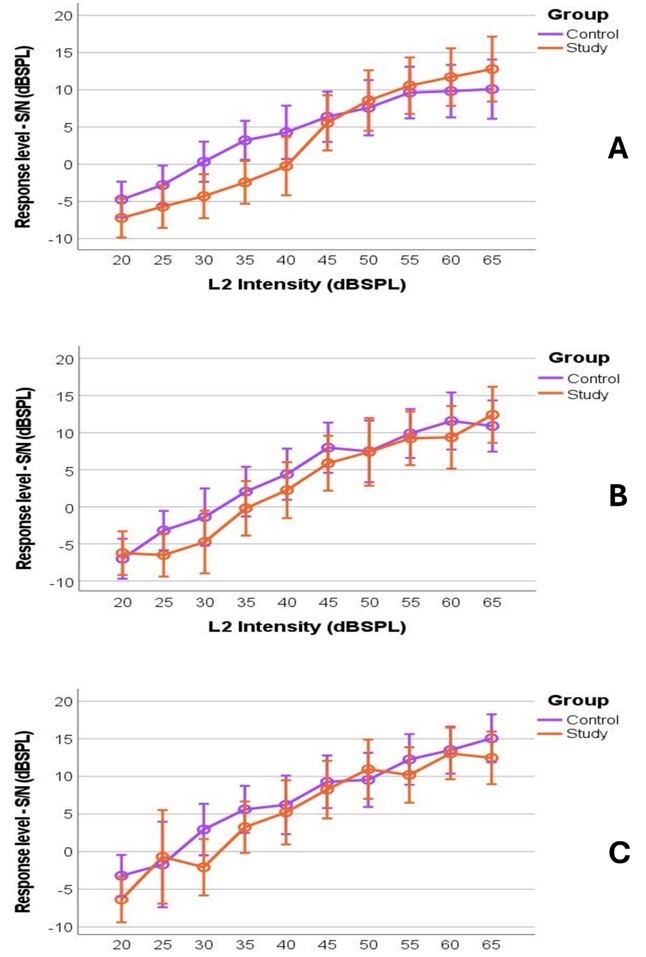
DPOAE Growth Curves. Representation of the means for the signal-to-noise ratio (S/N) of the DPOAE recorded by the Control and Study Groups in relation to the intensity level of the L2 stimulus ranging from 20 to 65 dBSPL. (A) L2 = 2000Hz; (B) L2 = 3000Hz; (C) L2 = 4000Hz

The response pattern for the growth curve associated with the tested frequencies of 2000, 3000 and 4000 Hz can be seen in Figure 1, comparing both groups studied, respectively.

There were no statistically significant differences between the groups (control and study) regarding the responses (signal/noise ratio) of the DPOAE for all intensities at the frequency of 2000, 3000 and 4000 Hz.

**Figure 2:**
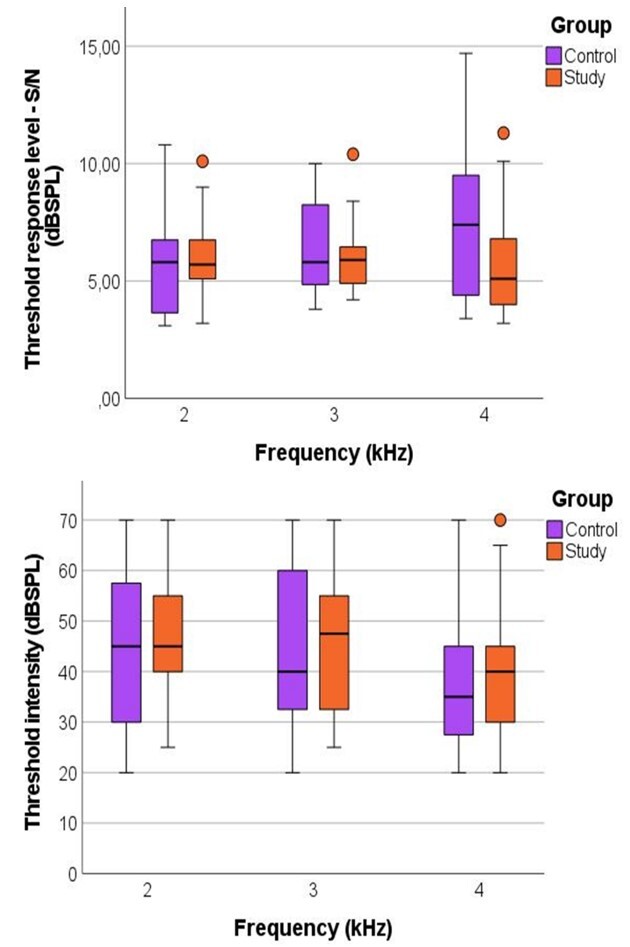
Top Panel: Threshold response level according to the L2 frequency for the Control and Study groups. Lower Panel: L2 intensity at the threshold according to the L2 frequency for the Control and Study groups

The measures of central tendency and dispersion of the stimulus’ response level and intensity at the threshold according to group and frequency can be visualized in Figure 2.

**Figure 3:**
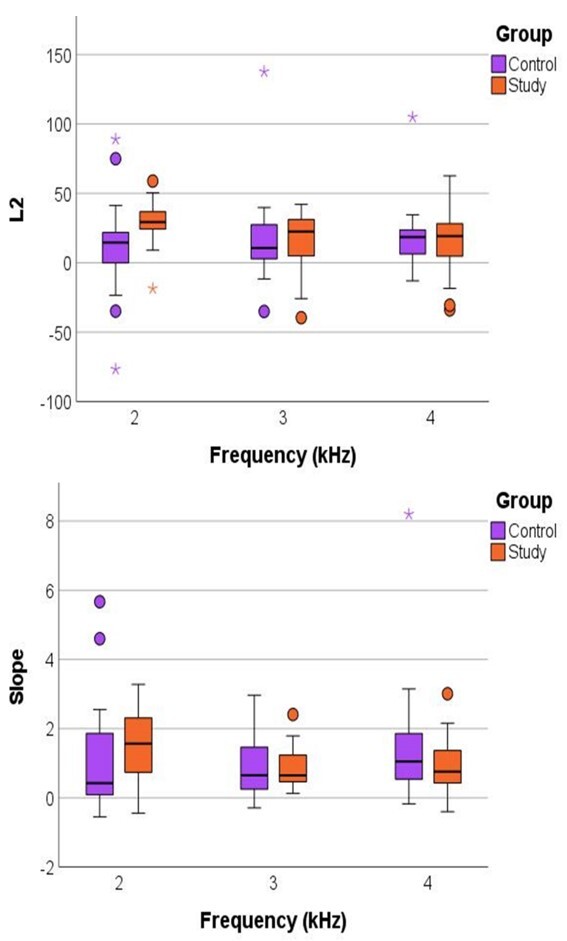
(A) Threshold Value (L2) according to the frequency for the control and study groups; (B) Slope of the growth curve according to the L2 frequency for the control and study groups. Note: some outlier values were not included in the graph scale

The L2 measurements and slope of the DPOAE growth curve according to the studied groups and frequency are shown in Figure 3.

There was a significant difference between the groups in relation to L2 and slope for the 2000 Hz frequency, where, for both parameters, the SG presented a higher value than the CG. No statistically significant differences were observed between the groups for the remaining parameters and frequencies

## DISCUSSION

This is one of the first studies to investigate the occurrence of discrete cochlear impairments that, despite not affecting the responses of a conventional audiogram, may still be present in people (schoolchildren), impairing temporal ordering tasks. Due to the novelty of this still recent topic of interest, it was not possible to find many studies addressing cochlear function with similar procedures in students.

A disorder in the cochlear nonlinearity/compression mechanism would alter the cochlea’s gain function, impairing the amplification (and perception) of low-intensity sounds and hindering speech perception^(6,13)^. This impairment in the cochlear amplification rate can alter the temporal auditory processing, given there may be deficits in the identification of sound characteristics, such as frequency, intensity and duration, especially for sounds that occur in a short period of time^(24)^. With this in mind, it was interesting to analyze the cochlear compression through the DPOAE growth curve in two groups of schoolchildren with normal audiograms, but differing in terms of performance in temporal auditory tests.

In the present study, the individuals of both the control group and the study group did not present statistically significant differences when compared in relation to age, gender and hearing thresholds. To this extent, the participants did not exhibit peripheral alterations and thus fit this research’s initial criteria, according to which the groups would differ only in terms of the temporal ordering tests results. Hunter et al.^(25)^ investigated the occurrence of peripheral alterations in children with idiopathic learning disorders. The study’s findings indicated that the DPOAE responses (signal-to-noise ratio), although less robust in the group of children presenting alterations, did not indicate statistical differences when compared to the typical development group, consequently, cochlear differences between the two groups were not confirmed. Such results corroborate the assumption that it would not be possible to distinguish these groups through the conventional audiological test battery. Hunter et al.^(25)^ further suggested that the identification of subtle impairments associated with the peripheral auditory function requires procedures that are not included in the basic audiological evaluation battery. Hence, it is important to assess the DPOAE growth curves, since it is a tool that allows the detection of subtle cochlear alterations^(6)^.

In the growth curves analysis of both groups, it was found a discrepancy regarding the intensity of the stimulus where the DPOAE threshold (L2) occurs, as well as in the slope comparison of the DPOAE growth curve for the 2000 Hz frequency. These parameters are related to cochlear nonlinearity^(10,17)^. The study group recorded a higher DPOAE threshold than the control group, indicating that children with alterations in temporal ordering tests require a greater intensity to reach the DPOAE threshold at a 2000 Hz frequency, when compared to children without any complaints. The increase in the slope value at 2000 Hz may also imply a more linear cochlear amplification, evidencing a cochlear compression loss for the presented stimuli^(10,26)^.

The abovementioned result can be better assimilated in the context of the cochlear compression concept. Cochlear compression is involved in the cochlear amplification rate and is characterized by a higher amplification for low-intensity stimuli while providing a lower amplification for high- and medium-intensity stimuli. Compression is essential to listening processes in unfavorable environments as well as to the perception of rapid changes in stimuli, being part of the temporal processing^(13,27,28)^.

Children with altered temporal processing skills may exhibit difficulties in understanding speech, instructions, along with a poor sound localization ability^(29)^. One possible explanation has to do with the temporal aspect of auditory information processing, in which speech understanding is influenced by time, being associated with the temporal processing ability (divided into temporal ordering, temporal resolution, temporal integration and masking). Amplitude-modulated signals are useful for speech recognition and are related to the way the auditory system processes temporal cues^(30)^. Slow fluctuations in the speech envelope across different spectral channels are known to carry auditory information relevant to speech intelligibility. The temporal auditory characteristics processed by the auditory system, with its onset on the cochlea, are a crucial for speech intelligibility^(30,31)^. Moreover, preserved skills in the temporal auditory processing allow the individual to identify subtle acoustic differences in speech^(32)^.

The growth curve at 2000 Hz divided the SG from the CG. The presence of a difference between the groups’ cochlear function instigates a line of investigation on the peripheral function integrity’s contribution to the improvement of auditory information, from the peripheral portion up to the auditory center. In this regard, further studies are necessary, incorporating longitudinal research to assess the response alteration over time^(25)^, considering the prospect of different emergence thresholds for the DPOAE. Another suggestion is to use other procedures to investigate the temporal resolution, such as the silence interval identification tests (including the Gaps In Noise - GIN), that evaluate the minimum time required to identify and resolve acoustic events, contrasting with the temporal ordering tests performed herein, which are related to the processing of stimuli and ordering them accordingly, following the correct way in which they were presented.

Further stimulus paradigms have been used to measure the DPOAE growth functions. The scanning paradigm proposed by researchers^(33)^ has been proving itself as an efficient method to evaluate DPOAE growth, as it can produce almost continuous growth functions at multiple frequencies. Additional research using different DPOAE recording methods and probes would enable a promising assessment of the cochlear function in the investigated group.

The present study is motivated by the investigation of the relationship between deficits in temporal ordering tests and the cochlear function, combined with the consideration of whether the cochlear gain analysis by DPOAE growth function measurement could be feasible in children. Identifying potential differences in the cochlear function of children characterized by the presence or absence of alterations in temporal tests justifies this research subject matter. Therefore, the temporal ordering ability was isolated for examination, since the auditory processing encompasses a series of auditory skills that are not all simultaneously altered, prompting a complex profile with distinct and not always related complaints.

By studying temporal processing specifically, which the literature establishes as related to the cochlear function, it becomes possible to determine a line of reasoning for the assessment of the peripheral hearing influence on the temporal auditory processing. Nevertheless, it is probable that this approach may convey certain research limitations, since there is a possibility that the children of both groups present other auditory skills impairments. The results of this study should be interpreted under this perspective. Thus, different aspects of the peripheral hearing still remain under question, motivating the continuation of the research on cochlear function and its repercussion on auditory behavior in schoolchildren.

## CONCLUSION

Given the abovementioned study limitations, it can be concluded that, according to the sample investigated herein, children with alterations in temporal tests required a greater intensity to reach the DPOAE threshold at the 2000 Hz frequency, when compared to children without complaints. Furthermore, the participants presented a more linear cochlear amplification at this same frequency, as indicated by the increase in the slope value. Such cochlear dysfunctions, even if subtle, can influence the perception of temporal sound patterns.
