# Supplementary material for: DPOAE growth function in schoolchildren with impaired temporal ordering skills
Source: Codas. 2025 Feb 10;37(2):e20240071. doi: 10.1590/2317-1782/e20240071en (PMC11813184; doi:10.1590/2317-1782/e20240071en)
Supplement: Translation

# Translation: Função de crescimento das EOAPD em escolares com prejuízo em habilidade auditiva de ordenação temporal

**DOI:** 10.1590/2317-1782/e20240071pt

**Authors:** Patricia Kimiko Kumagai, Seisse Gabriela Gandolfi Sanches, Renata Mota Mamede Carvallo

**Affiliations:** 1 Faculdade de Medicina, Universidade de São Paulo – USP - São Paulo (SP), Brasil.; 2 Departamento de Fisioterapia, Fonoaudiologia e Terapia Ocupacional, Faculdade de Medicina, Universidade de São Paulo – USP - São Paulo (SP), Brasil.

**Keywords:** Emissões Otoacústicas Espontâneas, Percepção Auditiva, Testes Auditivos, Audição, Criança

## Abstract

**Objetivo:**

Investigar se as respostas cocleares de um grupo de crianças com testes de ordenação temporal dentro da normalidade seriam distintas daquelas encontradas em crianças com tais testes alterados.

**Método:**

Participaram do estudo 25 crianças entre 8 e 13 anos, com limiares audiométricos normais, timpanometria tipo A e emissões otoacústicas-produto de distorção (EOAPD) presentes em ambas as orelhas. Destas, 13 crianças formaram o Grupo Estudo e 12 o Grupo Controle. O Grupo Estudo se diferenciou do Grupo Controle por apresentar alteração em testes auditivos temporais. Além dos testes para averiguar os critérios de inclusão, foi realizado o registro da curva de crescimento das EOAPD para três diferentes frequências f2, respectivamente 2002, 3003 e 4004 Hz. Os estímulos foram apresentados na intensidade de f2 (L2) de 20 a 65 dBNPS em degraus de 5 dB e o nível de intensidade de f1 (L1) seguiu a fórmula: L1=0,4L2+39 dB. Os dados foram tratados estatisticamente, adotando-se nível de significância de 5%.

**Resultados:**

Os grupos não se diferenciaram em relação aos valores das EOAPD convencionais (DP-Grama). O Grupo Estudo diferenciou-se do Grupo Controle por exibir tanto o limiar (p=0,034), como o slope mais elevado (p=0,043) nas curvas de crescimento de EOAPD, isoladamente na frequência de 2000Hz.

**Conclusão:**

Crianças com alteração em testes de ordenação temporal necessitam de uma maior intensidade para alcançar o limiar das EOAPD na frequência de 2000 Hz quando comparadas às crianças sem queixas, apresentando também uma amplificação coclear mais linear nessa mesma frequência, indicada pelo aumento do valor do slope.

## INTRODUÇÃO

Um dos procedimentos que visam a avaliar a função das células ciliadas externas (CCE) presentes no Órgão de Corti, é a medida das emissões otoacústicas evocadas^(1)^. As emissões otoacústicas podem ser evocadas por diferentes estímulos, os quais geram também diferentes respostas que podem auxiliar o diagnóstico audiológico e a identificação de discretas alterações na função coclear^(2-4)^.

As emissões otoacústicas produto de distorção (EOAPD) são geradas a partir da apresentação de dois tons puros de diferentes frequências, denominadas f1 e f2, sendo a frequência de f2 mais alta que f1. A resposta coclear é o produto da distorção dos dois estímulos de tons puros, sendo mais comumente usado na prática clínica o produto de distorção que corresponde à 2f1-f2^(2)^.

A presença das EOAPD sugere função coclear preservada e, por outro lado, sua ausência requer investigação cuidadosa de orelha média e orelha interna^(5,6)^. Essas emissões são fundamentais em testes para auxiliar na identificação de perdas auditivas de grau moderado a profundo na população infantil, minimizando os impactos negativos da uma perda auditiva em relação ao desenvolvimento da linguagem e alfabetização^(2)^.

O registro das EOAPD é obtido com o emprego de pares de tons puros apresentados a um nível de intensidade fixo para F1 e F2, geralmente 65dBNPS/55 dBNPS respectivamente. No exame, o conjunto das respostas para os pares de tons puros gera um gráfico (DP-grama) com as respostas da cóclea para os pares de tons puros investigados, normalmente entre 1000 e 6000Hz em intensidade fixa.

Contudo, além das respostas cocleares para intensidades fixas nas diferentes frequências, também é de interesse clínico saber qual a menor intensidade de estímulo (F1 e F2) que evoca uma emissão otoacústica robusta^(7-9)^. Isso porque, mesmo que diferentes cócleas apresentem resposta de DP-grama semelhantes, elas podem ter diferentes limiares de EOAPD. Ou seja, diferentes cócleas podem responder de forma semelhante para nível de intensidade média (65/55 dB) mas apresentarem limiares de EOAPD diferentes. Esta condição pode ser útil em diferenciar aspectos da função coclear entre pessoas que embora tenham limiares auditivos dentro da área de resposta normal, apresentem diferentes queixas auditivas. Para investigar o limiar de EOAPD é possível apresentar níveis de intensidade de F1 e F2 de forma crescente ou, decrescente, entre 20 e 70 dBNPS, para identificar a menor intensidade de estímulo capaz de gerar uma EOAPD robusta^(10,11)^. Desta forma, é possível registrar o limiar, bem como determinar a curva de crescimento da resposta de EOAPD em relação ao crescimento do estímulo. É a denominada Curva de Crescimento de entrada/saída, que fornece informações tanto sobre o limiar das EOAPD, quanto sobre os mecanismos cocleares associados à função de não linearidade das CCE^(11,12)^. Na função coclear normal, os mecanismos não lineares fornecem maior taxa de amplificação para estímulos de baixo nível de intensidade, e assim aumentam a sensibilidade e a seletividade de frequências, características da função auditiva normal^(13)^. Os mecanismos não-lineares promovem a compressão da amplificação coclear à medida que o estímulo aumenta^(13,14)^, ou seja, diminuem a taxa de amplificação para sons de média intensidade. Esta amplificação não-linear estabelece mecanismos que contribuem para alta sensibilidade, a larga faixa dinâmica e a sintonia fina do sistema auditivo em mamíferos^(15)^.

Os conceitos de “não-linearidade” e de “compressão” na função coclear estão intimamente ligados. Cócleas saudáveis mantêm uma amplificação linear para estímulos de baixo nível de intensidade, diminuem a amplificação para estímulos de média intensidade (compressão), e aumentam, novamente, para estímulos de alto nível de intensidade. A redução na amplificação para estímulos de média intensidade está relacionada à taxa de compressão do ganho coclear. As EOAPD permitem a investigação da taxa de compressão do amplificador coclear por meio de suas curvas de crescimento de entrada/saída, sendo essa compressão medida por meio da inclinação da curva de crescimento (*slope)* ^(13)^. Estudos têm mostrado redução na taxa de compressão com o avanço da idade, evidenciado por *slope* mais linear com o avanço da idade^(7,11,16)^.

O limiar das Curvas de Crescimento de EOAPD é designado como o menor nível de intensidade de L_2_ que gera uma resposta robusta. Limiares de EOAPD de nível de intensidade mais elevado podem estar relacionados à diminuição de resposta globais de EOAPD visualizadas no DP-grama^(5,7,17)^.

O processamento auditivo temporal está relacionado à aptidão do indivíduo em identificar as alterações nas características dos sons, como a frequência, intensidade, duração e as pausas entre os estímulos. A percepção auditiva requer um processamento preciso da estrutura do tempo sonoro. Uma das tarefas envolvidas no processamento auditivo está relacionada à percepção de mudanças rápidas no padrão do estímulo e à integração das informações que ocorrem ao longo do tempo. Esta percepção é denominada de processamento temporal. As habilidades envolvidas no processamento temporal são: ordenação temporal, integração temporal, mascaramento temporal e resolução temporal^(18)^. A ordenação temporal é responsável por identificar dois ou mais estímulos apresentados em uma ordem e em um determinado período, estando relacionada também à compreensão da informação sonora^(18)^. Alguns pesquisadores sugerem que os resultados insatisfatórios em leitura estejam relacionados com um comprometimento auditivo do processamento temporal, sendo difícil para as crianças ouvirem alterações acústicas dos sons, o que pode levar a uma percepção inadequada da fala^(19)^ e dificuldades na consciência fonológica. Ao estudarem crianças com alterações de leitura e escrita, Soares et al.^(20)^ observaram uma associação entre alteração de processamento temporal e déficit na consciência fonológica.

Considerando-se que ligeiras alterações cocleares identificadas pelas curvas de crescimento afetam a resolução temporal, uma das subclassificações do processamento temporal^(6)^ torna-se relevante avaliar se, nas dificuldades de ordenação temporal, também é possível identificar discretas alterações cocleares.

Uma vez que as curvas de crescimento das EOAPD podem ser uma ferramenta a mais para a identificação de discretos comprometimentos da função coclear^(7)^, pode ser útil na investigação da função auditiva periférica em crianças com alteração em testes auditivos temporais.

A partir da hipótese de que o desempenho em testes temporais auditivos possa ser afetado por alteração na taxa de compressão de função coclear, o objetivo deste estudo é investigar se as respostas cocleares de um grupo de crianças com testes de ordenação temporal dentro da normalidade seriam distintas daquelas encontradas em crianças com tais testes alterados.

## MÉTODO

Este estudo foi aprovado pelo Comitê de Ética em Pesquisa Institucional, em 24/09/2019, segundo o Parecer: 3.631.330 e CAAE: 20319119.0.0000.0065. Todos os participantes do estudo assinaram Termo de Consentimento Livre Esclarecido e o Termo de Assentimento Informado aprovando a participação na pesquisa.

A amostra deste estudo foi de conveniência composta por 25 crianças, entre 08 e 13 anos, sendo 12 pertencentes ao grupo Controle (5 do sexo feminino e 7 do sexo masculino) e 13 pertencentes ao grupo Estudo (6 do sexo feminino e 7 do sexo masculino). A média de idade para o grupo Controle foi de 9,8 (±1,34) anos e no grupo Estudo 10,5 (±1,71) anos. Foram critérios de inclusão: limiares tonais até 20 dBNA nas frequências de 250 a 8000 Hz; respostas presentes de EOAPD; timpanograma Tipo A com sonda de 226Hz. Para crianças do grupo Estudo foi também critério de inclusão a presença de prejuízo em processamento auditivo temporal, comprovado pela alteração no teste de padrão de frequência e/ou no teste de padrão de duração. Já as crianças do grupo Controle não apresentaram alterações em ambos os testes de ordenação temporal, sendo adotado o padrão de normalidade para crianças brasileiras falantes de português^(21)^. Foram excluídas crianças que apresentassem transtorno de atenção ou hiperatividade diagnosticados ou queixa e/ou diagnóstico de alterações neurológicas ou psiquiátricas.

Todos os participantes foram submetidos à inspeção do meato acústico externo; timpanometria e pesquisa de reflexos acústicos (ipsi e contralaterais) com o equipamento AT 235h (Interacoustics). Em seguida, a audiometria tonal via aérea foi realizada no audiômetro GSI 61 (Grason Stadler), com fones TDH 50P. Foram testadas, pelo método ascendente-descendente, as frequências de 0.25 a 8 kHz, em intervalos de oitavas. Após a verificação dos critérios de inclusão, os participantes realizaram os testes temporais (versão infantil, Auditec Saint Louis^(22)^) Padrão de Frequência (TPF) e Padrão de Duração (TPD).

Após a bateria de testes comportamentais, as Emissões Otoacústicas Evocadas – produto de distorção (EOAPD) foram coletadas pelo equipamento ILO 292II V6 OAE Otodynamics, com o paciente acomodado em cabina acústica.

Dois métodos foram realizados na obtenção das EOAPD: DP Grama e Curvas de crescimento:

**A-** Para obtenção do DP grama, os estímulos f1 e f2 foram apresentados ao nível de intensidade fixa L1 e L2 de 65 dB e 55 dB respectivamente e variação de frequências de f2 de 1001 a 6006 Hz (2 pontos por oitava), sendo f2/f1 aproximadamente 1,22. Foram consideradas presentes as respostas com relação Sinal/Ruído maior que 6 dB NPS em relação ao desvio padrão do ruído de fundo.
**B-** Três curvas de crescimento de EOAPD foram traçadas para cada orelha, selecionando-se uma única frequência de F2 para cada uma das curvas na função DP Growth (curva de crescimento) do programa do ILO V6. Foram realizados os registros da curva de crescimento de EOAPD e a determinação do limiar de EOAPD para as três diferentes frequências de f2, respectivamente 2002, 3003, 4004 Hz. Os estímulos foram apresentados na intensidade de estímulo f2 (L2) variando de 20 a 65 dB NPS em degraus de 5 dB e o nível de intensidade do estímulo f1 (L1) variou segundo a fórmula proposta por Kummer et al.^(23)^: L1= 0,4L2+39 dB. A regra utilizada para interromper o teste foi determinada quando o assoalho de ruído de fundo ficou abaixo de 10 µPA, em todas as intensidades de estímulo pesquisadas e as respostas das EOAPD estavam estabilizadas (quase sem variação entre as varreduras). O limiar das EOAPD foi considerado como a menor intensidade em que houve relação sinal/ruído ≥ 03 dB com as duas intensidades superiores sequentes apresentando maior ou igual relação sinal/ruído. O grau de inclinação da curva foi avaliado pelo *slope* (medida gerada pelo software) com o objetivo de verificar a compressão coclear.

Na realização de todos os testes foi considerado o conforto da criança, o teste foi interrompido em qualquer desconforto ou cansaço apresentado.

Para o cálculo dos intervalos de confiança de 95% foi utilizado o método de viés corrigido e acelerado com base em 2000 amostras *bootstrap*. Os valores entre colchetes nas tabelas indicam os limites superior e inferior dos intervalos de confiança de 95%.

Foram avaliadas as respostas das EOAPD (relação sinal/ruído), e, no perfil das curvas de crescimento, o limiar (L2), e o *slope*. Para comparação entre os grupos, foi utilizado o teste t de *Student* para amostras independentes (paramétrico) e U de *Mann-Whitney* (não-paramétrico), quando preservado o pressuposto de normalidade da distribuição dos dados (p > 0,05, teste de normalidade de Shapiro-Wilk). Nos casos do uso do teste *t de Student* para amostras independentes nos quais foi observada violação do pressuposto de homoscedasticidade (p ≤ 0,05, teste de Levene), utilizou-se a correção de *Welch* para heteroscedasticidade no cálculo do valor de p. O tamanho do efeito da diferença entre os grupos foi medido por meio do cálculo do coeficiente d ou r. O valor de significância estatística adotado foi igual a 5% (p ≤ 0,05). Utilizou-se o software *SPSS Statistics*, versão 25.0 (IBM Corp., Armonk, NY, EUA).

### Resultados

Este estudo analisou as respostas cocleares de crianças, quanto ao limiar e à configuração da curva de crescimento de EOAPD por meio da medida de *slope* disponibilizada pelo software do ILO 292 II - V6 (Otodynamics) em escolares com e sem alterações em testes temporais auditivos.

A análise estatística dos dados do estudo foi realizada usando como base a amostra de 50 orelhas organizadas em dois grupos: Grupo Controle (GC, n = 24) e Grupo Estudo (GE, n = 26).

Não houve diferença significativa entre as idades do grupo (p=0,320). Também não houve diferença significativa entre os grupos em relação aos limiares auditivos para todas as frequências entre 250 e 8000 Hz.

Os resultados da Tabela 1 demonstram que não houve diferença estatisticamente significante entre os grupos em relação ao limiar dos reflexos de 500 a 4000 Hz. Sendo assim, indivíduos com alteração em testes de ordenação temporal apresentaram limiares para reflexos acústicos semelhantes aos de indivíduos sem alteração em testes de ordenação temporal.

**Tabela 1:** Valores descritivos e análise comparativa dos grupos em relação aos limiares dos reflexos acústicos

**Frequência (Hz)**	**Grupo**	**n**	**Média**	**DP**	**Mediana**	**Mín.**	**Máx.**	**P**	**T.E.**
500	GC	24	93,33	6,70	95,00	75,00	105,00	0,708	0,055
			[90,42, 96,04]		[95,00, 95,00]				
	GE	24	94,17	7,32	95,00	80,00	105,00		
			[91,32, 96,88]		[95,00, 95,00]				
1000	GC	24	92,71	8,84	95,00	75,00	110,00	0,587	0,165
			[89,38, 95,83]		[87,50, 95,00]				
	GE	24	94,17	9,63	95,00	75,00	110,00		
			[90,42, 97,92]		[92,50, 97,50]				
2000	GC	24	91,67	9,96	90,00	75,00	110,00	0,341	0,139
			[87,71, 95,83]		[90,00, 92,50]				
	GE	24	94,58	11,41	95,00	80,00	110,00		
					[95,00, 95,00]				
			[90,00, 98,96]						
4000	GC	24	97,29	7,07	100,00	80,00	105,00	0,971	0,008
			[94,38, 100,00]		[95,00, 100,00]				
	GE	24	96,04	9,89	100,00	75,00	105,00		
			[92,08, 100,00]		[95,00, 102,50]				

Legenda: GC = Grupo Controle; GE = Grupo Estudo; DP = Desvio padrão; Mín. = Mínimo; Máx. = Máximo; P = p valor; T.E. = Tamanho do efeito

Na Tabela 2 são apresentadas as medidas de tendência central e de dispersão da resposta (relação sinal/ruído) das EOAPD convencionais de acordo com o grupo e com a frequência.

**Tabela 2:** Valores descritivos e análise comparativa dos grupos em relação à resposta das EOAPD convencionais de acordo com a frequência

**EOAPD**	**Grupo**	**n**	**Média**	**DP**	**Mediana**	**Mín.**	**Máx.**	**P**	**T.E.**
1001 (S/R – dB)	GC	24	2,38	9,87	2,55	-16,80	20,10	0,291	0,278
			[-1,28, 6,01]		[-0,10, 6,65]				
	GE	26	5,12	8,27	5,65	-10,40	20,60		
			[2,06, 7,97]		[1,05, 7,75]				
1501 (S/R – dB)	GC	24	11,35	11,07	11,00	-9,00	34,00	0,947	0,018
			[7,37, 15,42]		[5,05, 15,85]				
	GE	26	11,55	10,23	12,60	-15,30	28,50		
			[7,60, 15,21]		[6,10, 16,20]				
2002 (S/R – dB)	GC	24	11,68	11,63	13,45	-15,00	32,20	0,518	0,148
			[6,94, 15,97]		[8,20, 16,60]				
	GE	26	13,40	5,89	13,45	1,70	25,40		
			[11,24, 15,44]		[11,55, 15,20]				
3003 (S/R – dB)	GC	24	13,03	10,83	13,45	-15,90	29,00	0,547	0,087
			[8,23, 17,10]		[11,40, 19,10]				
	GE	26	13,40	5,70	12,90	2,60	25,60		
			[11,24, 15,44]		[10,85, 15,30]				
4004 (S/R – dB)	GC	24	17,79	7,22	19,15	5,30	30,00	0,337	0,252
			[15,13, 20,59]		[15,55, 20,10]				
	GE	26	15,97	6,03	16,85	2,60	26,60		
			[13,77, 18,17]		[13,90, 20,10]				

Legenda: GC = Grupo controle; GE = Grupo estudo; S/R = relação sinal/ruído; DP = Desvio padrão; Mín. = Mínimo; Máx. = Máximo; P = p valor; T.E. = Tamanho do efeito

Não houve diferença significativa entre os grupos em relação à resposta das EOAPD convencionais (Tabela 2).

O padrão de resposta para a curva de crescimento em relação às frequências testadas 2000, 3000 e 4000 Hz comparando ambos os grupos estudados, respectivamente, podem ser observados na Figura 1.

**Figura 1:** Curvas de Crescimento da EOAPD. Descrição das médias dos Grupos Controle e Estudo para a relação sinal/ruído (S/R) das EOAPD em relação ao nível de intensidade do estímulo L2 entre 20 e 65 dBNPS. (A) L2 = 2000Hz; (B) L2 = 3000Hz; (C) L2 = 4000Hz

Não houve diferenças estatisticamente significantes entre os grupos (controle e estudo) em relação às respostas (relação sinal/ruído) das EOAPD para todas as intensidades na frequência de 2000, 3000 e 4000 Hz.

As medidas de tendência central e de dispersão do nível de resposta e intensidade do estímulo no limiar de acordo com o grupo e a frequência podem ser visualizadas na Figura 2.

**Figura 2:** Painel Superior: Nível de resposta no limiar de acordo com a frequência de L2 para os grupos Controle e Estudo. Painel Inferior: Intensidade de L2 no limiar de acordo com a frequência de L2 para os grupos Controle e Estudo

As medidas de L2 e s*lope* da curva da curva de crescimento das EOAPD de acordo com os grupos estudados e a frequência estão dispostas na Figura 3.

**Figura 3:** (A) Valor Limiar (L2) de acordo com a frequência para os grupos controle e estudo; (B) Slope da curva de crescimento de acordo com a frequência de L2 para os grupos controle e estudo. Nota: alguns valores *outliers* estão fora da escala do gráfico

Houve diferença significativa entre os grupos em relação a L2 e *slope* para a frequência de 2000 Hz, sendo que, para os dois parâmetros, o GE apresentou maior valor em comparação ao GC. Para os demais parâmetros e demais frequências, não foram observadas diferenças estatisticamente significantes entre os grupos.

## DISCUSSÃO

Este é um dos primeiros estudos a investigar a ocorrência de discretos comprometimentos cocleares que, embora não afetem as respostas em um audiograma convencional, podem estar presentes em pessoas (escolares) com prejuízo nas tarefas de ordenação temporal. Por ser um tema de interesse ainda recente, não foram encontrados muitos estudos que abordem a função coclear com procedimentos semelhantes em escolares.

O prejuízo no mecanismo de não-linearidade/compressão coclear alteraria a função de ganho da cóclea, prejudicando a amplificação (e a percepção) de sons de baixo nível de intensidade e dificultando a percepção da fala^(6,13)^. Este prejuízo na taxa de amplificação coclear pode alterar o processamento auditivo temporal, pois pode haver prejuízo na identificação das características dos sons, tais como frequência, intensidade e duração, principalmente para sons que ocorrem em um curto período de tempo^(24)^. Assim, foi interesse do estudo analisar a compressão coclear por meio da curva de crescimento das EOAPD, em dois grupos de escolares com audiograma dentro da normalidade, mas diferindo quanto ao desempenho em testes temporais auditivos.

No presente estudo, os participantes tanto do grupo controle quanto do grupo estudo, quando comparados em relação à idade, sexo e limiares auditivos, não apresentaram diferenças estatisticamente significantes. Desta forma, não exibiram alterações periféricas e encaixaram-se nos critérios iniciais da pesquisa, segundo os quais os grupos se diferenciariam apenas quanto aos resultados dos testes de ordenação temporal. Hunter et al.^(25)^ investigaram a ocorrência de alterações periféricas em crianças com distúrbios idiopáticos de aprendizagem. Os resultados do estudo mostraram que respostas de EOAPD (relação sinal/ruído), embora menos robustas no grupo de crianças com alteração, não apresentaram diferenças estatísticas em comparação ao grupo de desenvolvimento típico, não sendo assim confirmadas diferenças cocleares entre os dois grupos estudados. Estes resultados mostram que na bateria audiológica convencional não seria possível distinguir os grupos. Hunter et al.^(25)^ sugeriram ainda que a identificação de comprometimentos discretos de função auditiva periférica requer procedimentos não inseridos na bateria audiológica básica. Por este motivo ressalta-se a importância de avaliar as curvas de crescimento das EOAPD pois é uma ferramenta que possibilita a detecção de alterações cocleares sutis^(6)^.

Na análise das curvas de crescimento entre os grupos houve diferença na intensidade do estímulo onde ocorre o limiar das EOAPD (L2) e na comparação do *slope* da curva de crescimento das EOAPD para a frequência de 2000 Hz. Estes dois parâmetros são relacionados a não linearidade coclear^(10,17)^. O grupo estudo apresentou limiar mais elevado das EOAPD em comparação ao grupo controle, indicando que crianças com alteração em testes de ordenação temporal necessitam de uma maior intensidade para alcançar o limiar das EOAPD na frequência de 2000 Hz, quando comparadas a crianças sem queixas. O aumento do valor do *slope* em 2000 Hz pode também indicar uma amplificação coclear mais linear, apontando déficit na compressão coclear para os estímulos apresentados^(10,26)^. O resultado acima pode ser compreendido à luz do conceito de compressão coclear. A compressão coclear está envolvida na taxa de amplificação coclear e é caracterizada por maior amplificação para estímulos de baixa intensidade e menor amplificação para os de alta e média intensidade. A compressão tem papel nos processos de escuta em ambientes desfavoráveis e na percepção de mudanças rápidas nos estímulos, fazendo parte do processamento temporal^(13,27,28)^.

Crianças com habilidade de processamento temporal alterada podem ter dificuldades para entender a fala, instruções, e também ter a habilidade de localização sonora prejudicada^(29)^. Uma das explicações está associada ao aspecto temporal do processamento da informação auditiva, no qual a compreensão da fala é influenciada pelo tempo, estando relacionada com a habilidade de processamento temporal (dividida em ordenação temporal, resolução temporal, integração temporal e mascaramento). Sinais de fala modulados em amplitude são úteis para o reconhecimento de fala e estão relacionados com a forma como o sistema auditivo processa as pistas temporais^(30)^. Sabe-se que as flutuações lentas no envelope da fala em diferentes canais espectrais carregam informações auditivas relevantes para a inteligibilidade de fala. As características auditivas temporais processadas pelo sistema auditivo, desde a cóclea, são um ponto crítico para inteligibilidade de fala^(30,31)^. Habilidades preservadas em processamento auditivo temporal possibilitam que o indivíduo identifique pequenas diferenças acústicas na fala^(32)^.

A curva de crescimento em 2000 Hz separou GE e GC. A presença de diferença na função coclear entre os grupos abre uma linha de investigação sobre a integridade da função periférica contribuindo para o aprimoramento da informação auditiva, da porção periférica para a central auditiva. Nesse sentido, o prosseguimento dos estudos se faz necessário, incluindo pesquisas longitudinais que permitam avaliar a mudança da resposta ao longo do tempo^(25)^ podendo haver uma diferença nos limiares de surgimento das EOAPD. Outra sugestão é o emprego de outros procedimentos que investiguem a resolução temporal, tais como testes de identificação de intervalos de silêncio (dentre eles o *Gaps In Noise*- GIN) que avaliam o tempo mínimo necessário para identificar e resolver os eventos acústicos, e que diferem dos testes de ordenação temporal, realizados neste estudo, os quais estão relacionados ao processamento dos estímulos e assim ordená-los na forma correta em que foram apresentados.

Outros paradigmas de estímulo vêm sendo utilizados para medir a função de crescimento das EOAPD. O paradigma de varredura proposto por pesquisadores^(33)^ tem surgido como um método eficiente para medir o crescimento das EOAPD, pois pode produzir funções de crescimento quase contínuas em múltiplas frequências. Outras pesquisas utilizando outros paradigmas e sondas de captação de EOAPD possibilitariam uma avaliação promissora da função coclear no grupo investigado.

Este é um estudo motivado pela indagação sobre a relação entre prejuízo em testes de ordenação temporal e função coclear, além de investigar se a análise do ganho coclear por medidas de função de crescimento das EOAPD poderia ser viável em crianças. Identificar eventuais diferenças na função coclear entre grupos de crianças diferenciados pela presença ou não de alteração em testes temporais, justifica essa linha de investigação. Desta forma, isolamos a habilidade de ordenação temporal para estudo, uma vez que o processamento auditivo engloba uma série de habilidades auditivas e nem todas estão alteradas simultaneamente, mostrando um quadro complexo, com queixas distintas e nem sempre relacionadas. Ao estudar especificamente o processamento temporal, que a literatura aponta como relacionada à função coclear, possibilita traçar uma linha de raciocínio para a investigação da influência da audição periférica no processamento auditivo temporal. No entanto, é possível que esta escolha implique em certas limitações do estudo, uma vez que não está excluída a possibilidade de que outras habilidades auditivas estejam comprometidas em crianças de ambos os grupos. Os resultados do presente estudo devem ser interpretados sob esse foco. Assim, diferentes facetas da audição periférica ainda permanecem sob indagação, motivando o prosseguimento das pesquisas sobre função coclear e sua repercussão no comportamento auditivo em escolares.

## CONCLUSÃO

Tendo em vista as limitações do estudo apresentadas acima, podemos concluir que, na amostra investigada no presente estudo, as crianças com alteração em testes temporais necessitaram de uma maior intensidade para alcançar o limiar das EOAPD na frequência de 2000 Hz quando comparadas às crianças sem queixas. Além disso, apresentaram amplificação coclear mais linear nessa mesma frequência, indicada pelo aumento do valor do *slope*. Tais disfunções cocleares, mesmo sendo discretas, podem exercer influência na percepção de padrões temporais do som.
